# *Oplopanax horridus*: Phytochemistry and Pharmacological Diversity and Structure-Activity Relationship on Anticancer Effects

**DOI:** 10.1155/2018/9186926

**Published:** 2018-09-13

**Authors:** Kai Wu, Chong-Zhi Wang, Chun-Su Yuan, Wei-Hua Huang

**Affiliations:** ^1^Department of Physiatry, Xiangya Hospital, Central South University, Changsha 410008, China; ^2^Tang Center for Herbal Medicine Research, The Pritzker School of Medicine, University of Chicago; 5841 South Maryland Avenue, MC 4028, Chicago, IL 60637, USA; ^3^Department of Clinical Pharmacology, Xiangya Hospital, Central South University, Changsha 410008, China

## Abstract

*Oplopanax horridus*, well-known as Devil's club, is probably the most important ethnobotanical to most indigenous people living in the Pacific Northwest of North America. Compared with the long history of traditional use and widespread distribution in North America, the study of* O. horridus* is relatively limited. In the past decade, some exciting advances have been presented on the phytochemistry and pharmacological diversity and structure-activity relationship on anticancer effects of* O. horridus*. To date, no systematic review has been drafted on the recent advances of* O. horridus*. In this review, the different phytochemicals in* O. horridus* are compiled, including purified compounds and volatile components. Animal and in vitro studies are also described and discussed. Especially, the potential structural-activity relationship of polyynes on anticancer effects is highlighted. This review aimed to provide comprehensive and useful information for researching* O. horridus* and finding potential agents in drug discovery.

## 1. Introduction

*Oplopanax horridus* (Sm.) Torr. & A. Gray ex Miq., well-known as Devil's club, an ethnobotanical, exclusively originates and is distributed in northwestern North America. This understory shrub forming larger populations through layering usually grows in moist, well-drained, dense, and old-growth forests [1]. This herbal plant belongs to the genus* Oplopanax*, which only consists of three species, the remaining of which are* O. japonicus* Nakai and* O. elatus* (Nakai) Nakai [2].* O. elatus* is mainly distributed in the temperate regions of Northern China, the south of Primorye, northern part of the Korean Peninsula, and Russia [3], while* O. japonicus* is endemic to central Japan [4]. The genus* Oplopanax* is classified to the Araliaceae family, which comprises some world-known botanicals such as* Panax ginseng* (Asian ginseng, Chinese or Korean ginseng),* P. quinquefolius* (American ginseng), and* P. notoginseng* (Sanqi) [5–8]. Occasionally,* O. horridus* is sometimes marketed as “wild armored Alaskan ginseng,” “Alaskan ginseng,” or “Pacific ginseng”, but the marketing is now forbidden in the United States due to the misleading [2]. Such marketing relies on the presumable speculation that* O. horridus* share similar chemical ingredients with the herbs in* Panax*, but the presumption is not supported by related phytochemical investigation [4].

*O. horridus* has a long history of use for 34 different medical ailments by the Pacific indigenous peoples from over 38 linguistic groups [7]. The extracts of Devil's club are marketed in North America for respiratory stimulant and expectorant, rheumatoid arthritis, autoimmune conditions, eczema, type II diabetes, external infections, and internal infections [7]. The Pacific native tribes also use* O. horridus* for spiritual stimulant, while Shaman use it in their religion ceremonial practices.  Table 1 summarizes that different part of* O. horridus* is used for many various ailments by the indigenous people. It implies that the main phytochemicals in different parts of* O. horridus* are significantly varied.

Recently, pharmacological studies showed that* O. horridus* possessed anticancer, antibacterial, antidiabetes, antipsoriasis, antiarthritis, and antifungal and anticonvulsant activities [2]. In the past five years, most of animal and in vitro studies on* O. horridus* focused on its anticancer effects. At the same time, extensive investigation on the chemical constituents of* O. horridus* has led to the isolation and identification of compounds** 1-47**, which include polyynes (polyacetylenes), phenylpropanoids (aglycones and glycosides), lignan glycosides, triterpenoids, sesquiterpenes, and some other compounds. Additionally, forty-eight volatile compounds were identified from the volatile oil by GC/MS. Among these components, polyynes, e.g., falcarindiol (FAD), and oplopantriol A (OPT), have been mostly reported as potentially anticancer natural products from* O. horridus*.

In the past decade, some exciting advances have been presented on the phytochemistry, pharmacological diversity, and structure-activity relationship on anticancer effects of* O. horridus*. To date, no systematic review has been drafted on the recent advances of* O. horridus*. In this review, the different phytochemicals in* O. horridus* are compiled, including purified compounds and volatile components. Animal and in vitro studies are also described and discussed. Especially, the potential structural-activity relationship of polyynes on anticancer effects is highlighted. This review aimed to provide comprehensive and useful information for researching* O. horridus* and finding potential agents in drug discovery.

## 2. Ethnobotanical and Ethnopharmacology

**Figure 1:**
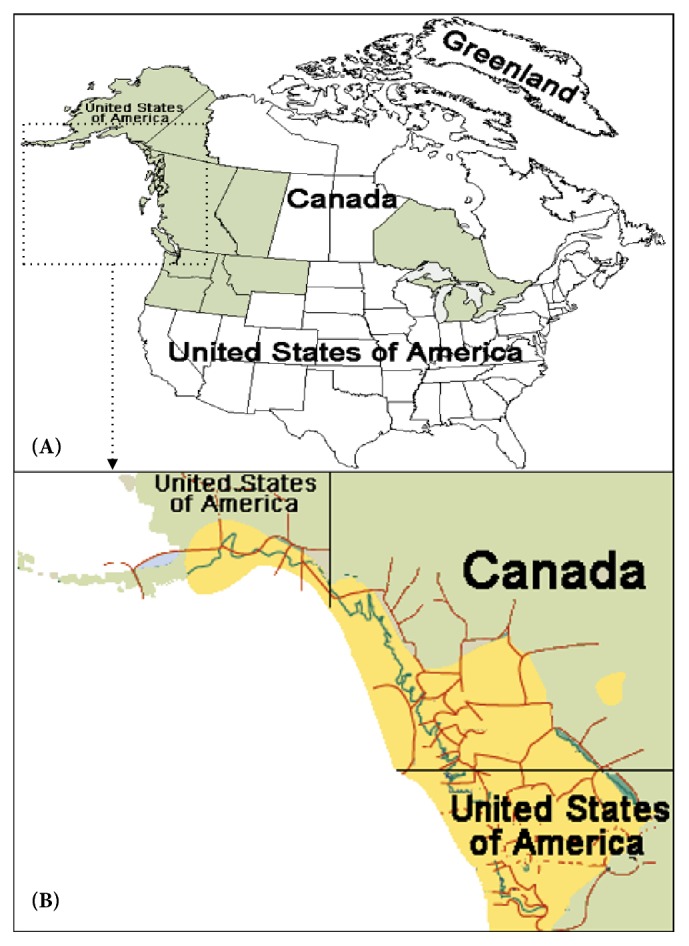
Geographic distribution of* O. horridus.*

*O. horridus* is distributed from Alaska along the Pacific Coast down to Oregon, Idaho, and Montana in the south and east to the southwestern Yukon Territory, while some isolated populations grow around Michigan and the islands in Lake Superior (Figure 1) [7, 9].* O. horridus* generally is from 1 to 1.5 m tall, while it can grow to 3 to 5 m in some undisturbed moist rainforest. The spines appear around the stems and along the upper and lower surfaces of its leaves, which are palmately lobed with 5-13 lobes, 20 to 40 cm across, and are spirally located on the stems (up to 3 cm diameter). The flowers are blooming in dense umbels from 10 to 20 cm diameter with five greenish-white petals. The fruit is a small red drupe with 4 to 7 mm diameter. The root without spines is similar to the stem [9]. The plant grows and reproduces slowly and take many years to reach seed bearing maturity, and it is very sensitive to human impact [10].

*O. horridus* has been used for both spiritual and medical practices by the native tribes of Alaska and British Columbia for centuries. Traditionally, the native tribes drink the aqueous decoction of the root or stem bark of* O. horridus* for the treatments of colds, fever, burns, stomach trouble, body pains, sore throats, swollen glands, constipation, and tuberculosis [11]. The inner bark of the root and stem has been used to stop infection on wounds and reduce swelling [12]. The Alaska Natives from the Alaskan southeast coast, Cook Inlet, Kodiak, Kenai, Prince William Sound, and Tanana Valley use the inner bark of the root and stem to treat colds, cough, and fever [13]. The traditional preparations of* O. horridus* include either applying the heated inner bark to the wounded area and bandaging it or chewing the root or stem bark and spitting the crude herb on the wound as an emergency analgesic and antiseptic [10]. The infusion of inner bark of* O. horridus* was described as a possible treatment for cancer by indigenous peoples in North America [7]. Although formal documentation of its traditional use for cancer is lacking,* O. horridus *extracts and its compounds exhibited the anticancer activities in preclinical investigations [2, 4].

## 3. Phytochemistry

The main chemical constituents of* O. horridus *are polyynes (polyacetylenes), phenylpropanoids (aglycones and glycosides), lignan glycosides, triterpenoids, sesquiterpenes, and volatile compounds [4]. Within the different structural skeletons of these groups, a variety of analogues are presented in the aerial and underground parts of the botanical. Generally, the secondary metabolites in* O. horridus* are consistent with the patterns of chemical constituents from the genus* Oplopanax* and the family* Araliaceae*. Polyynes have been mostly reported with high contents in both the stem and the root of* O. horridus* as potential anticancer and antimycobacterial (antituberculosis) natural products. Due to the limited number of analytical chemistry investigations that have been done, only six polyynes have thus far been quantified in the root of* O. horridus* [14, 15]. Phenylpropanoids and lignan glycosides are the other two main natural products from the genus* Oplopanax*. Sesquiterpenes and volatile compounds have also been extracted from the root of this herb. For this species, the chemical profile of the polyynes of the root may be useful for herbal identification since they are very different from the other species in this genus,* O. japonicus* and* O. elatus*.

### 3.1. Polyynes (Polyacetylenes)

Polyynes, also known as polyacetylenes, possess a long chain of carbon atoms with alternating double and triple bonds between them, which occur in natural products containing more than two acetylene groups. Meanwhile, polyynes comprising two to four acetylene groups are usually found in higher plants, most of which contain conjugated diyne in the highly unsaturated lipid chains [16]. These highly unsaturated hydrophobic compounds are generated in the* Araliaceae* family, which have a high concentration in the roots and a small content in the stem barks. The polyynes which are unstable and oxidized in dried plant material, contain seventeen or eighteen carbons (C17 or C18) in the skeleton chain, and are mostly found in the* Araliaceae*.

**Figure 2:**
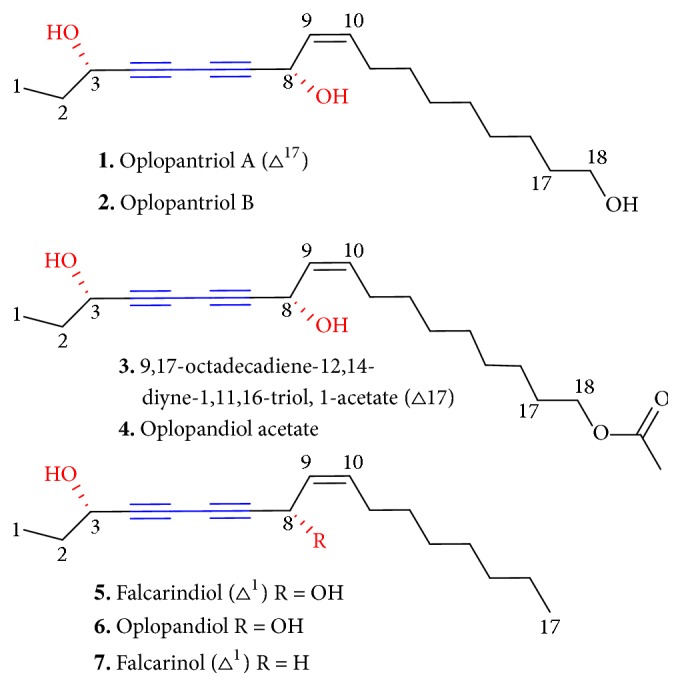
Polyynes of* O. horridus*.

To date, seven polyynes have been purified and identified from the root bark of* O. horridus*, five of which are also discovered from the stem bark of* O. horridus* [17, 18]. The identified C17 and C18 polyynes from* O. horridus*,** 1–7**, are oplopantriol A, oplopantriol B, (11*S*, 16*S*, 9*Z*)-9,17-octadecadiene-12,14-diyne-1,11,16-triol,1-acetate, oplopandiol acetate, falcarindiol, falcarinol, and oplopandiol (Figure 2). Polyyne has not been reported to be found in the follower, berry, leaves, and seeds of* O. horridus*. For analytical investigation of polyynes in* O. horridus*, an HPLC fingerprint method was developed to evaluate the stem and berry extracts of* O. horridus*, which showed that polyynes are not major components in the stem and berries of* O. horridus* [19]. For the quantification of polyynes in* O. horridus*, an online solid-phase extraction HPLC (SPE-HPLC) and a modified reversed migration microemulsion electrokinetic chromatography were validated to quantify polyynes and one polyene in* O. horridus* [14, 15]. The contents of polyynes in the root bark of* O. horridus* range from 0.029% to 0.15% (**1**), 0.025% to 0.13% (**2**), 0.077% to 0.31% (**3**), 0.24% to 0.43% (**4**), 0.26% to 0.67% (**5**), and 0.26% to 0.67% (**6**), respectively.

Although no evidence is found that* O. horridus* is used to cure cancer in North America in the long history, the extracts and polyynes from* O. horridus* exhibited their anticancer activities in recent pharmacological research. The extracts mainly containing polyynes from* O. horridus* root bark showed effective in the inhibition of the proliferation of several ovarian, breast, lung, and colorectal cancer cell lines [20, 21]. The mechanisms of polyynes on anticancer activities are still not well known. To the tested polyynes, Falcarindiol and Oplopantriol A showed the strongest inhibition on the growth of all the examined cancer cell lines [22, 23].

### 3.2. Phenylpropanoids (Aglycones and Glycosides)

Phenylpropanoids are metabolized by plants from the amino acids phenylalanine and tyrosine. The basic chemical structure of phenylpropanoid consists of the aromatic phenyl group and the three-carbon propene tail of cinnamic acid. Phenylpropanoids are found commonly in the plant kingdom, where they play a vital role as essential components in biosynthesizing polymers, providing protection from ultraviolet light, defending against herbivores and pathogens, and mediating plant-pollinator interactions. Phenylpropanoids including aglycones and glycosides were also obtained from* O. horridus* [24]. As phenolic compounds, phenylpropanoids and their derivatives were synthesized into thousands of different chemical structures including aromatic rings in several species in the* Araliaceae* family.

**Figure 3:**
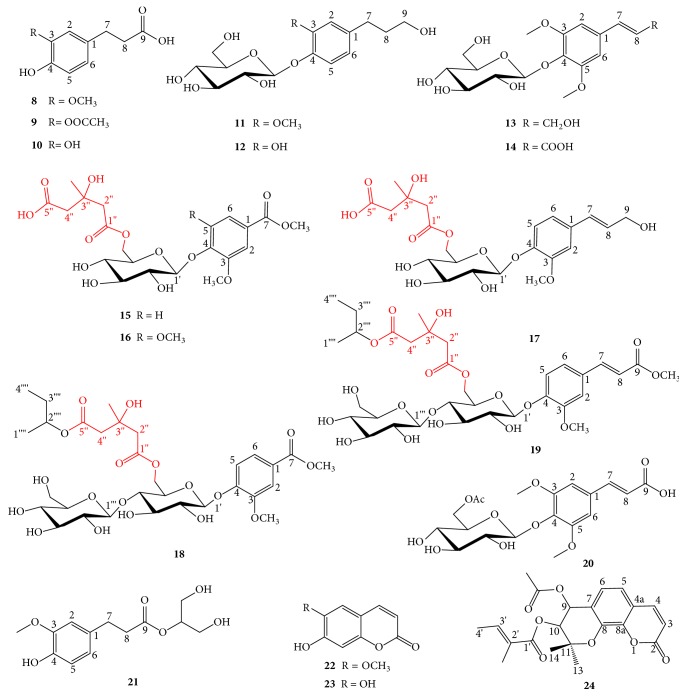
Phenylpropanoids of* O. horridus.*

The phenylpropanoids from the root bark of* O. horridus *were isolated and purified by various chromatographic techniques including silica gel, reverse phase silica gel, sephadex LH-20, and pre-HPLC and identified by their physicochemical properties and spectral data. The phenylpropanoids are obtained and identified from the root bark of* O. horridus* include ferulic acid (**8**), 3-acetylcaffeic acid (**9**), caffeic acid (**10**), homovanillyl alcohol 4-O-beta-D-glucopyranoside (**11**), 3-hydroxyphenethyl alcohol 4-O-beta-D-glucopyranoside (**12**), 3, 5-dimethoxycinnamyl alcohol 4-O-beta-D-glucopyranoside (**13**), 3, 5-dimethoxycinnamyl alcohol 4-O-beta-D-glucopyranoside (**14**), oplopanphesides A-E (**15-19**), 3-{4-[(6-O-acetyl-beta-D-glucopyranosyl) oxy]-3,5-dimethoxyphenylpropanoic acid (**20**), and glycer-2-yl ferulate (**21**), as well as three coumarins, scopoletin (**22**), esculetin (**23**), and 3′-angeloyl-4′-acetyl-cis-knellactone (**24**) (Figure 3) [25–27]. Phenylpropanoids (**15–19**) are glycosides that possess a novel feature in their sugar moieties with a 3-hydroxy-3-methylglutaryl moiety connected with C-6 of the beta-D-glucopyranosyl group.

Although the phenylpropanoids are only discovered from the root bark of* O. horridus* several years ago, no phenylpropanoids have been qualitatively and quantitatively determined by any analytical method. Therefore, the contents of the phenylpropanoids in any part of* O. horridus* are still unknown. These phenylpropanoids showed no cytotoxic effects against human cancer cell lines (HCT-116, HT-29, MDA-231, and MCF-7) by MTT method [26]. Phenylpropanoids have not been found in other parts of* O. horridus*, while some phenylpropanoids are recently found in the root of* O. elatus* [28].

### 3.3. Lignan Glycosides

The lignans are a large group of natural products found in plants, which have also been obtained from the family Araliaceae. Usually, plant lignans are derived from phenylpropanoids via dimerization of substituted cinnamic alcohols, which is catalysed by oxidative enzymes and controlled by dirigent proteins. Due to the rarely investigation on the hydrophobic constituents from* O. horridus*, no lignan aglycone has been found in* O. horridus*. Meanwhile, lignan glycosides are firstly purified and identified from the hydrophilic constituents of the root bark of* O. horridus* [25, 27].

**Figure 4:**
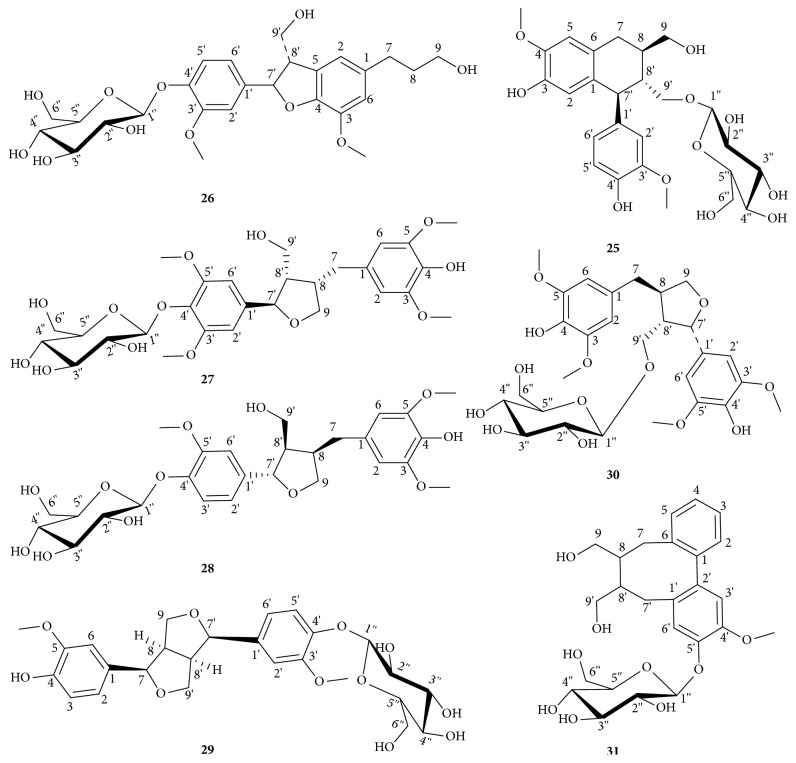
Lignan glycosides of* O. horridus.*

The isolated lignan glycosides from the root bark of* O. horridus* are identified as (+)-isolaricires-inol-9′-O-beta-D-glucopyranoside (**25**), 3, 3′-dimethoxy-4, 9, 9′-trihydroxy-4′, 7-epoxy-5′, 8-lignan-4, 9-bis-O-beta-D-glucopyranoside (**26**), (+)-5, 5′-dimethoxylariciresinol 4′-O-beta-D-glucopyranoside (**27**), (-)-5,5′-dimethoxylariciresinol 4′-O-beta-D-glucopyranoside (**28**), (-)-pinoresinol 4′-O-beta-D-glucopyranoside (**29**), (+)-5, 5′-dimethoxylariciresinol 9′-O-beta-D-glucopyranoside (**30**), and (+)-[5,6,7,8-tetrahydro-7-(hydroxylmethyl)-10,11-dimehoxydibenzo[*a*,* c*][8]annulen-6-yl]methyl beta-D-glucopyranoside (**31**) (Figure 4). To date, lignan glycoside has not yet been found in other parts of* O. horridus*, perhaps due to the limited phytochemical study of these parts. No analytical method has been developed to determine lignans in* O. horridus* so the contents of lignan glycosides in* O. horridus* are unclear. Little pharmacological research has been reported on the lignan glycosides from* O. horridus*, so the corresponding bioactivities of these lignan glycosides are yet to be determined.

### 3.4. Triterpenoids

Triterpenoids including saponins are the most representative constituents of the family Araliaceae, which have also been found in the genus* Oplopanax*. Except one dammara-type triterpenoid, all other triterpenoids belong to oleanane- and lupan-type, which are all only discovered in the leaves of the herbs in this genus. Unlike ginseng from the genus* Panax*, triterpenoid has neither been isolated nor identified from the root of the plants in* Oplopanax*, but triterpenoids are regarded to be the main bioactive substances in ginseng. Recently, one dammara-type triterpenoid and five lupan-type triterpenoids have been isolated and identified from the leaves of* O. horridus* [29, 30].

**Figure 5:**
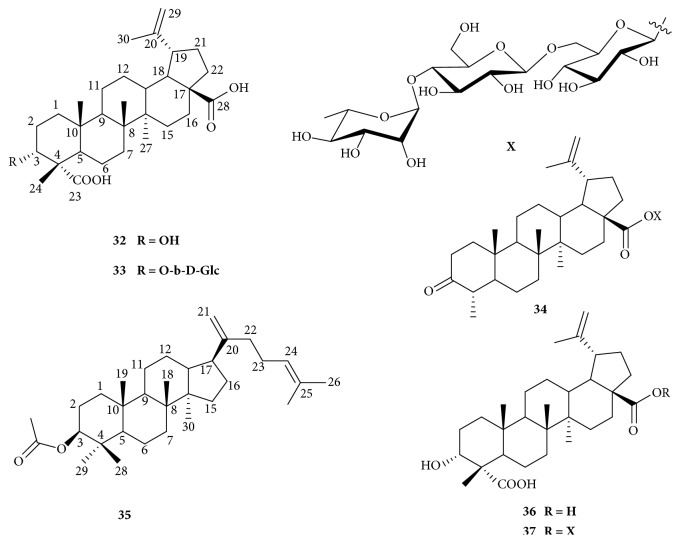
Triterpenoids of* O. horridus.*

The purified triterpenoids from the leaves of* O. horridus* are elucidated as 3*α*-hydroxy-lup-20(29)-ene-23, 28-dioic acid (**32**), 3-alpha-hydroxy-lup-20(29)-ene-23, 28-dioic acid-3-beta-*β*-D-glucopyranoside (**33**), 24-nor-3-oxo-lup-20(29)-en-28-oic acid-28-O-alpha-L-rhamnopyranosyl (1′′′ → 4′′)-beta-D-glucopyranosyl(1′′ → 6′)-beta-D-glucopyranoside (**34**), dammara-20, 24-dien-3beta-ol acetate (**35**), acankoreagenin (**36**), and acankoreoside A (**37**) (Figure 5). Although dammarane triterpenoid is very common in the family Araliaceae, especially in the genus* Panax*, this triterpenoid is the first time to be found in the family Araliaceae. Compared with 41 triterpenoids obtained from the leaves of* O. elatus*, only six triterpenoids isolated from* O. horridus* are limited. The contents of triterpenoids in the leaves of* O. horridus* are uncertain because of few reports about their determination. It is also blank about the pharmacological research on the triterpenoids from* O. horridus*.

### 3.5. Sesquiterpenes

**Figure 6:**
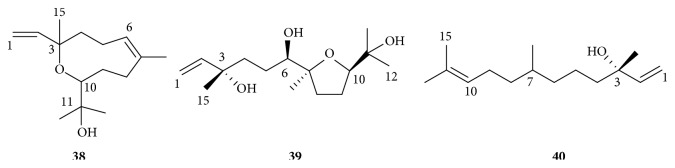
Sesquiterpenes of* O. horridus.*

Sesquiterpenes are a group of terpenes including sesquiterpenoids that consist of three isoprene units, which may be acyclic or contain rings, including many unique combinations. Sesquiterpenes are widely found in plants from Araliaceae, most of which were identified by gas chromatography coupled with mass spectrometry (GC/MS). To date, three sesquiterpenes have been purified from the inner stem bark and root bark of* O. horridus*, which were identified as 3,10-epoxy-3,7,11-trimethyldodeca-1,6-dien-11-ol (neroplomacrol,** 38**),* rel*-(3*S*,6*R*,7*S*,10*R*)-7,10-epoxy-3,7,11-trimethyldodec-1-ene-3,6,11-triol (neroplofurol,** 39**), and nerolidol (**40**) (Figure 6)[31]. Among them, neroplomacrol and neroplofurol were isolated from the anti-TB active fractions of the inner stem bark of* O. horridus*, which were not the main anti-TB active principles but may have synergistic behavior with them. Other sesquiterpenes were identified from the essential oil of* O. horridus* by GC/MS, the relative contents of which are also calculated.

### 3.6. Volatile Compounds

The volatile oil from* O. horridus* is regarded to be responsible for some clinical uses of the title plant. Volatile oils from natural medicines are very complicated, and volatile compounds could not be easily purified and obtained from volatile oil by modern chromatographic techniques due to their disadvantages such as lower boiling point, lower polarity, and thermal degradation. Therefore, most volatile compounds are identified before by GC/MS. Volatile oils have been extracted from stem and root of* O. horridus*.

Shao et al. used a supercritical fluid extraction method to extract the volatile oil from the root bark of* O. horridus* with a yield of 1.15%, which was much more than the yield of 0.22% when the oil was extracted by the steam distillation method. Then, the volatile oil was subsequently analyzed by GC/MS (Table 2)[32, 33]. Forty-eight volatile compounds were identified by GC/MS analysis and, among all the detectable constituents, (*S*,* E*)-nerolidol (52.5%) was the compound with highest content in the volatile oil, followed by *τ*-cadinol (21.6%) and bicyclogermacrene (4.5%). (*S*,* E*)-Nerolidol and *τ*-cadinol had been purified as simple compounds from the essential oil and their structures were identified by additional spectroscopic techniques. By comparison with the MS data from the Agilent Chemstation the library, one more polyyne,* S*-falcarinol (3.6%), was also detected and obtained from the essential oil by chromatographic separation. Except for some very common volatile compounds, e.g., *α*-pinene, *β*-phellandrene, linalool, 1,3,5-undecatriene, and 1,3,5,8-undecatetraene; most of the constituents identified in the essential oil were sesquiterpenes and oxygenated sesquiterpenes. Some minor constituents were not identified by GC/MS.

### 3.7. Other Compounds

Some fatty acids, steroid, and steroid glycosides were separated and identified from the root bark of* O. horridus*, i.e., heptacosane acid (**41**), ethacosane acid (**42**),* beta*-sitosterol (**43**), daucosterol (**44**), aralia cerebroside (**45**), usnic acid (**46**), and protocatechoic acid (**47**) [24].

## 4. Biological Activities

Different parts of* O. horridus* are used to help cope with verified illnesses from type II diabetes to cancer problems by indigenous people, but the pertinent pharmacological researches are very limited. Compared to few biological activity report of the leaves, the extracts of the stem and berries have only been screened for their antiproliferation effects of four human cancer cell lines (SW-480, HCT-116, HT-29, MCF-7, and NSCLC)[19]. Meanwhile, the root has been researched on its antibacterial, antifungal, antidiabetes, and anticancer effects [2, 34]. In the past decade, although no record was found that any part of* O. horridus* was used to cure cancer in the long history, the extracts and compounds from the root of* O. horridus* exhibited their anticancer activities in recent investigations [2, 4].

### 4.1. Antibacterial

The extracts and fractions from the root of* O. horridus* showed antibacterial activity related with* antimycobacterial*, the bacteria of which may cause leprosy and tuberculosis in humans [35–37]. The results showed that the ingredients of* O. horridus* displayed synergistic enhancement on antituberculosis effect [38]. From a methanol extract of the inner bark of* O. horridus* exhibited antibacterial activity, five polyynes,** 1–4**,** 7**, including falcarinol, falcarindiol, oplopandiol, oplopandiol acetate, and (*Z*)-9,17-octadecadiene-12,14-diyne-1,11,16-triol 1-acetate, are purified and identified. Specifically, falcarindiol and oplopandiol were the main positive antimycobacterial components from the extract [17]. Additionally, all the obtained polyynes displayed potential ability to inhibit the proliferation of* M. tuberculosis*,* M. avium* at 10 *μ*g/disk in a disk diffusion assay, as well as two Gram-positive bacteria,* Bacillus subtilis* and* Staphylococcus aureus*, two Gram-negative bacteria,* Escherichia coli* DC2 and* Pseudomonas aeruginosa* Z61, and the yeast* Candida albicans*. Among them, falcarindiol has been proved to possess the strongest antibacterial activities [17].

### 4.2. Antifungal

The methanol extract of* O. horridus* inner bark was screened for antifungal activity against 9 fungal species, i.e.,* Aspergillus flavus, Aspergilfus fumigatus, Candida albicans, Fusarium tricuictum, Microsporum cooker, Microsporum gypseum, Saccharomyces cerevisiae, Trichoderma viridae, *and* Trichophyton mentagrophytes* [39, 40]. The extract was demonstrated antifungal activity against all the fungal strains assayed except* A. flavus*. The extract had only slight activity against* A. fumigatus, C. albicans, F. tricuictum, S. cerevisiae, *and* T. viridae*, but it had greater inhibition against* M. gypseum*. With the assayed fungi, the extract showed the greatest significant antifungal activity on* M. cooker* and* T. mentagrophytes* [39]. However, the corresponding natural products with antifungal activity are still unknown.

### 4.3. Antidiabetes

*O. horridus* is extensively recommended for the treatment of diabetes in indigenous communities [41, 42]. Without any data, it was claimed that* O. horridus*, as a pancreatic tonic, is regarded to decrease blood sugar levels by improve the efficiency of insulin [41]. Therefore, it should be cautiously used for diabetes due to the perspective risk and uncertainty. In preclinical research on* O. horridus* for adult diabetes, a white precipitate isolated from root bark of* O. horridus* showed a slightly hypoglycemic effect in lab hares [43]. Inconsistently, the* O. horridus* tea at a certain dosage exhibited no significant hypoglycemic effects in a primary study [44]. Three new phenolic glycosides obtained from the root bark of* O. horridus*, oplopanpheside A-C, displayed negative activity for their *α*-glucosidase inhibition (IC_50_>50 *μ*M, respectively) [26]. Unambiguously, more studies are requested in concerning its antidiabetic effects.

### 4.4. Anticancer Effect

The infusion of inner bark of O. horridus was described as a possible treatment for cancer by indigenous linguistic people including Alutiiq, Gitxsan, Haida, Tlingit, and Tsimshian [7]. To date,* O. horridus* has not been reported to have anticancer effects on human bodies, and no human clinical studies have been conducted on this herb. The extracts mainly containing polyynes from the root bark of* O. horridus* show anticancer effects on several colorectal, breast, lung, ovarian, pancreatic and acute myeloid leukemia cancer cell lfnes, and animal models [4, 45, 46].

#### 4.4.1. Colorectal Cancer

The different extracts from* Oplopanax* root, stem, berry, root bark, and their fractions were investigated in vitro for their potential antiproliferative effects on human HCT-116, HT-29, and SW-480 colorectal cancer cell lines [19–21, 47]. The results showed that the stem, root, root bark extracts, and lipophilic fractions of root bark possessed potent antiproliferative effects. The observation of the cell cycle distribution suggested that G2/M phase is arrested by the extracts. However, the hydrophilic fractions had much weaker effects on the apoptotic cells [20].

Five polyynes were isolated and evaluated as the bioactive compounds from hydrophobic fractions [47]. Among these polyynes, FAD and OPT showed significant potent effects, and the primary structure-activity analysis suggested that these anticancer activities are related to the ethylenic bonds and acylations in the structures [48, 49]. Firefly luciferase-tagged HCT-116 cells were inoculated into the flanks of athymic nude mice used as the animal model to evaluate the in vivo antitumor potential of FAD and OPT from* O. horridus* [48]. The results showed that the FAD and OPT treatment groups exhibit significantly decreased xenogeny imaging signal intensities when compared with the control group. Quantitative analysis revealed that the FAD and OPT significantly inhibited xenograft tumor growth after the administration of FAD and OPT [22, 23]. A rat small intestine epithelial cell line, IEC-6, is used to evaluate the safety of FAD and OPT. At concentrations of 1–20 *μ*M, FAD and OPT do not inhibit the IEC-6 cell growth. In contrast, human HCT-116 and HT-29 colorectal cancer cell growth was significantly inhibited at 15 *μ*M of FAD and OPT [23, 49]. Further study showed that FAD-induced cell death was mediated by the induction of endoplasmic reticulum (ER) stress and activation of the unfolded protein response (UPR). The FAD-induced ER stress and apoptosis was correlated with the accumulation of ubiquitinated proteins, suggesting that FAD functions, at least in part, by interfering with proteasome function, leading to the accumulation of unfolded protein and induction of ER stress [23].

In another study, OPT significantly suppressed malignant cells in both concentration- and time-dependent manner. The IC_50_ was approximately 5 *μ*M for HCT-116 and 7 *μ*M for SW-480 cells. OPT significantly induced apoptosis and arrested the cell cycle at the G2/M phase. The results showed that OPT significantly upregulated the expression of a cluster of genes, especially the tumor necrosis factor receptor family and the caspase family. The data suggested that the tumor necrosis factor-related apoptotic pathway played a key role in OPT-induced apoptosis [49].

The OPT-induced cancer cell death was also mediated by excessive ER stress. Decreasing the level of ER stress either by inactivating components of the UPR pathway or by expressing the ER chaperone protein GRP78 decreased OPT-induced cell death. OPT induced the accumulation of ubiquitinated proteins and the stabilization of unstable proteins, suggesting that OPT functions, at least in part, function by interfering with the ubiquitin/proteasome pathway [22].

#### 4.4.2. Acute Myeloid Leukemia (AML)

Male and female transgenic C57BL/6J-Foxp3-RFP mice (C57BL/6-Foxp3tm1Flv/J) were employed for in vivo anti-AML. After one week following engraftment, the 70% ethanol extract of* Oplopanax* root was diluted into the drinking water at a final concentration of 20 *μ*g/mL. The survival of both male and female mice was significantly increased upon the inclusion of the extract in the drinking water [45]. In addition, the extract decreased the Tregs and increased CD4^+^ T cells and the ratio of CD8^+^ T cells to Tregs. These changes are indicative of a responsive immune system correlated as expected with an increase in survival [45].

The 70% ethanol extract was assessed on AML cell line viability and to study the regulation of tyrosine phosphorylation and cysteine oxidation. The root extract displayed better in vitro anti-AML efficacy in addition to a noted anti-tyrosine kinase activity. The extract decreased the viability of murine C1498 cells and human U937, HL-60/VCR, and KG-1 cells with differential effects. In addition, the effect of the extract compared with nerolidol (a major essential oil component of* O. horridus*) yielded a better anti-AML activity [45].

#### 4.4.3. Breast Cancer

Effects of the extracts and polyynes have also been evaluated using MCF-7 and MDA-MB-231 human breast cancer cell lines [19, 21, 47]. The IC_50_ of the total extract, 50, 70, and 100% ethanol fractions on MCF-7 cells, were 248.4, 123.1, 44.0, and 31.5 *μ*g/mL, respectively [47]. The 100% ethanol fraction, which possessed the most potent anti-proliferative activity, showed the strongest apoptotic induction activity [19]. Additionally, three phenolic glycosides isolated from the hydrophilic fraction, oplopanphesides A, B, and C, showed no cytotoxic effects on MDA-MB-231 and MCF-7 cell lines [26]. Further, when the MCF-7 cells were treated with 30 *μ*g/mL of the total extract and fractions, the total extract increased the percentage of cells in the G_1_-phase to 49% and the decreased S phase to 6.2% [21].

#### 4.4.4. Lung Cancer

The antiproliferative effects of nonsmall cell lung cancer (NSCLC) cells were evaluated. The IC_50_ of the extract, 50, 70, and 100% ethanol fractions for antiproliferation on NSCLC cells, were 125.3, 271.1, 17.6, and 23.2 *μ*g/mL, respectively [19]. In addition, the extracts from the stem and berry were evaluated. The stem extract had potent antiproliferative effects at a low concentration of 0.1 mg/mL compared to 1 mg/mL for the berry [21].

#### 4.4.5. Ovarian Cancer

An ovarian cancer (Ovcar 10 three-dimensional [3D]) model was utilized to evaluate the antiproliferation activity of 75% ethanol extract and its active compound, alone and in combination. Ovcar 10 cells formed compact 3D spheroids after five days of culture in a rotary culture system [50]. The 3D spheroids were significantly more resistant to killing by the extract when compared to 2D cells. A number of apoptosis-related genes were differentially expressed in these cells. In 3D spheroids, the proportion of cells in the G2/M phase was slightly increased and the S phase was slightly decreased when compared to 2D cells [50].

#### 4.4.6. Pancreatic Cancer

The 70% ethanol extract was investigated on pancreatic endocrine HP62 and pancreatic ductal carcinoma PANC-1 and BxPC-3 cells. The extract significantly inhibited the proliferation of HP62 at low IC_50_. Apoptosis focused antibody array profile indicated upregulation of cytochrome C, claspin, cIAP-2 and HTRA2/Omi apoptosis-related markers, suggesting that the effect was via targeting the intrinsic mitochondrial apoptosis pathway [51].

The extract was also assessed for its effect on human pancreatic cancer PANC-1 3D spheroids and 2D monolayer cells. PANC-1 3D spheroids were significantly more resistant to killing by this extract with IC_50_ level closer to that observed in vivo. The extract also significantly enhanced the antiproliferation activity of the antineoplastic medications cisplatin (CDDP) and gemcitabine (GEM). The bioactive compound, identified as a polyyne, showed strong antiproliferation activities. Cell cycle analysis showed that the proportion of cells in S phase was increased and in G2/M phase was reduced in 3D spheroids when compared with 2D cells [52].

## 5. Structure-Activity Relationship of Polyynes on Anticancer Effects

### 5.1. Length of Carbon Chain

The structure-activity relationship of carbon chain in polyynes with their anticancer potential is summarized in Table 3. Anticancer activities decrease with the increase of the length of carbon chain in the main chemical structural skeleton of polyynes. To date, hundreds of polyynes have been isolated from bacterial, fungi, microorganisms, marine invertebrates, and high plants, and most naturally occurring polyynes feature a long lipid chain covering from 10 to 42 carbons in their chemical structural skeleton [16, 53, 54].

Among them, C_17_ and C_18_-polyynes were predominantly reported for their anticancer effects [55–58]. In the tested cancer lines, the antiproliferation effects of polyyne** 1 **displayed stronger effects than** 3** and** 5**, while polyyne** 2 **exhibited greater activities than** 4** and** 6** [48, 59]. Among these polyynes,** 1 **(FAD) shows the strongest anticancer activity, suggesting that FAD is a candidate for chemical modification [60–62].

### 5.2. Ethenyl Group

As shown in Table 3, (*R*)-falcarinol, a C_17_-polyyne, is obtained from some other plants, and it has been demonstrated to show antiproliferation effects on several cancer cell lines [63–67]. However, biological study has not been conducted on its derivative,* cis*-9-heptadecene-4,6-diyne-8-ol, which becomes an easier volatile compound when its terminal double bond is transferred to be single bond [68]. Regarding the chemical structures of these polyynes, they could be divided into three pairs grouped by their terminal bond moieties, i.e.,** 1** vs.** 2**;** 3** vs.** 4**; and** 5** vs.** 6**. With regard to their anticancer effects, polyyne** 1**,** 3**, and** 5 **showed greater potent activity, suggesting that the terminal ethenyl group improved anticancer activity [48, 59]. Recently, it is reported that the pharmacokinetic parameters between** 1** and** 2** had significant differences. Compared with** 2**, the terminal ethenyl group made** 1** easier transformed into other metabolites* in vivo* [69]. It has been demonstrated that compound** 1** could effectively join in chemical carcinogen detoxification by selectively mediating Phase II drug metabolism enzymes and quinone oxidoreductase [70]. Therefore, further studies should be employed to investigate the relationship between its terminal ethenyl group and anticancer behavior.

### 5.3. Hydroxyl Group

Since polar compounds interact with phospholipid substances in the hydrophilic domain of cell membrane, the entrance orientation of polyynes into membranes is affected by the number and location of alcohol hydroxyl groups [71]. The number and location of hydroxyl groups in polyynes influenced its anticancer activity [72, 73]. In the natural polyynes, if the hydroxyl group at C-1 was substituted with an acetyl group, the antiproliferative effect of polyyne was decreased, but if the hydroxyl group at C-11 or C-16 was acetylated, the antiproliferative activity would be significantly suppressed [59]. The anticancer activities of polyyne** 3**,** 4**,** 5**, and** 6** have been systematically compared. Oplopantriol A (**5**) showed the strongest antiproliferative effects on selected colorectal cancer cell lines and greater inhibition of tumor growth* in vivo *[22, 49]. Among the four polyynes, oplopantriol A can be considered to be a prochemical.

In order to investigate the contribution of hydroxyl groups to the antiproliferation on cancer cells, sixteen more acetylated derivatives have been synthesized for the evaluation of potential anticancer activities. The polyynes do not have any inhibitory effects of the adopted cancer cells if all the hydroxyl groups of the polyynes were acetylated [59]. However, compared to hydroxyl group at C-1, the terminal ethenyl group contribute more significance for maintaining anticancer potential. For example, compound** 5** has 3 to 5-fold relatively stronger antiproliferative effects than compound** 6**, while compound** 5 **possesses 1 to 3-fold relatively greater than compound** 3 **[48]. The elimination of hydroxyl groups of polyynes will yield acetylene alkanes, which have not been subjected to anticancer research.

### 5.4. Stereoselectivity

With the same planer chemical structures of the polyynes identified from* O. horridus*, each molecule is able to generate four stereoisomers with (3*S*, 8*S*)- or (11*S*, 16*S*)-, (3*S*, 8*R*)- or (11*R*, 16*S*)-, (3*R*, 8*S*)- or (11*S*, 16*R*)-, and (3*R*, 8*R*)- or (11*R*, 16*R*)-configuration. However, only (3*S*, 8*S*)- or (11*S*, 16*S*)- and (3*R*, 8*S*)- or (11*S*, 16*R*)-stereoisomers have been isolated and identified from natural resources [16]. According to the numbering sequence, the stereochemistry identified for polyynes purified from* O. horridus* with a (3*S*, 8*S*)- or (11*S*, 16*S*)-configuration seems to be very different from those with the (3*R*, 8*S*)- or (11*S*, 16*R*)-configuration reported from Apiaceae and Asteraceae [74–77].

To the (3*S*, 8*S*)- and (3*S*, 8*R*)-polyynes, (3*S*, 8*S*)-**1 **has only been found in the genus* Oplopanax*, while (3*S*, 8*R*)-**1 **has been isolated from several plants in different genus [78–81]. Although (3*R*, 8*S*)-**1 **and (3*R*, 8*R*)-**1** have been synthesized [77], they have not been assayed for their anticancer effects. (3*S*, 8*R*)-**1 **showed the inhibitory activity on various tumor cells proliferation, e.g., K562, Raji, Wish, HeLa, Calu-1, MCF-7* in vitro* [66, 82, 83], and tumor growth in different mouse model [67, 84]. Compared with (3*S*, 8*R*)-**1**, (3*S*, 8*S*)-**1** showed stronger antiproliferative effects against MCF-7 cells [23, 83]. Due to the limited data, the stereochemistry on the anticancer effects of** 1** needs further studies.

To the (11*S*, 16*S*)- and (11*S*, 16*R*)-polyynes, (11*S*, 16*R*)-**3 **has been isolated from the genus* Angelica* [85–87], while (11*S*, 16*S*)-**4 **has been synthesized [88, 89]. (11*S*, 16*R*)-**5** has also been isolated from the genus* Angelica* [90, 91], but (11*S*, 16*S*)-**6 **has only been found in* O. horridus*. Among these existing C_18_-polyynes, only (11*S*, 16*S*)-polyynes isolated from* O. horridus* have been tested for their anticancer activities. Therefore, more researches are needed to confirm the stereoselectivity of polyynes on their anticancer effects.

## 6. Summary and Perspectives

*O. horridus*, well-known as Devil's club, is probably the most important ethnobotanical to most indigenous people living in the Pacific Northwest of North America. Different part of* O. horridus* has a long history to be used for ailments by local people as traditional herbal medicines. In this review, the phytochemicals mainly including polyynes, triterpenoids, sesquiterpenes, diterpenoid, lignans, and phenylpropanoids from different part of* O. horridus* have been compiled. To date, approximately 47 compounds have been purified from* O. horridus*, and most of them are discovered for the first time from this genus. However, phytochemistry studies are relatively limited on each part of* O. horridus* except its root.

Polyynes identified from this herb exhibit pharmacological diversity. Especially, though this botanical has not been reported to have anticancer effects in human clinical studies, polyynes from* O. horridus* showed effects against colorectal cancer, breast cancer, lung cancer, ovarian cancer, pancreatic cancer, and acute myeloid leukemia in human cancer cell lines and different animal models. Regarding the anticancer effects and mechanisms of polyynes, it is pertinent to several molecular mechanisms and various signaling pathways. Because the bioavailability and metabolism of polyynes are critical important, their plasma concentrations that maybe significantly affect its biological activities* in vivo* are unknown. Moreover, the pharmacokinetic studies are blank to these polyynes except a few reported pharmacokinetic parameters of facarindiol and oplopandiol in rat [69]. Therefore,* in vivo* preclinical models are essential and vital to evaluate and predict the clinical function of polyynes.

We propose that the length of lipid chain, terminal ethenyl group, number of hydroxyl groups, and stereoselectivity in polyynes affect their anticancer activity. These relationships could benefit chemical modification of polyynes for discovery of bioactive molecules. Due to the stability of polyynes, C_17_-polyynes are most researched for their anticancer activities [61]. Our former experiments showed that acetylation of polyynes decreased their activities, but cancer chemopreventive potential may be enhanced by employing other chemical functional groups into polyynes. Although different stereoisomers of polyynes could be synthesized, their potential anticancer effects have not been quantitatively compared. Further investigations are needed to develop novel anticancer candidate in drug discovery.

## Figures and Tables

**Table 1:** The uses of *O. horridus* for various treatments.

Part of *O. horridus*	Application	Preparation	References
Inner barks	Respiratory, cardiovascular, gastrointestinal, cold or infection, diabetes, arthritis, and cancer	Decoction; infusion	[2, 7, 92]
Stems	Respiratory	Decoction	[2, 7, 93]
Roots	Respiratory, diabetes, and arthritis	Decoction; infusion	[2, 4, 94]
Berries	Gastrointestinal	Paste	[7, 19]
Leaves	Arthritis	Decoction; infusion	[7, 29]

**Table 2:** The volatile compounds of essential oil from the root of *O. horridus.*

Compound	Molecular formula	Compound	Molecular formula
Pentanol	C_5_H_12_O	Germacrene D	C_15_H_24_
Hexanol	C_6_H_14_O	ar-Curcumene	C_15_H_22_
Heptanal	C_7_H_14_O	*β*-Sesquiphellandrene	C_15_H_24_
Heptanol	C_7_H_16_O	*β*-Elemene	C_15_H_24_
Octanol	C_8_H_16_O	*α*-Muurolene	C_15_H_24_
Benaldehyde	C_7_H_6_O	Allo-aromadendrene	C_15_H_24_
2-Octenal	C_8_H_14_O	*γ* -Cadinene	C_15_H_24_
2-Methylpentenal	C_6_H_12_O	*α*-Farnesened	C_15_H_24_
*α*-Pinene	C_10_H_16_	1,10-Di-epi-cubenol	C_15_H_24_O
*β*-Phellandrene	C_10_H_16_	*δ* -Cadinene	C_15_H_24_
Linalool	C_10_H_18_O	Bicyclogermacrene	C_15_H_24_
1,3,5-Undecatriene	C_11_H_18_	Ishwarane	C_15_H_24_
1,3,5,8-Undecatetraene	C_11_H_20_	Germacrene B	C_15_H_24_
*α*-Ylangene	C_15_H_24_	(*S*, *E*)-Nerolidol	C_15_H_26_O
Aromadendrene	C_15_H_24_	Spathulenol	C_15_H_24_O
*α*-Zingiberene	C_15_H_24_	Germacrene D-4-ol	C_15_H_26_O
*β*-Caryophyllene	C_15_H_24_	Gleenol	C_15_H_26_O
(*E*)-*α*-Bergamotene	C_15_H_24_	Guaiol	C_15_H_26_O
*α*-Copaene	C_15_H_24_	Endo-1-bourbonanol	C_15_H_26_O
*α*-Humulene	C_15_H_24_	*τ*-Cadinol	C_15_H_26_O
*α*-Cadinene	C_15_H_24_	*τ*-Muurolol	C_15_H_26_O
Germacrene A	C_15_H_24_	*S*-Falcarinol	C_17_H_24_O
(*E*)-*β*-Farnesene	C_15_H_24_	*α*-Eudesmol	C_15_H_26_O
*γ* -Muurolene	C_15_H_24_	Bulnesol	C_15_H_26_O

**Table 3:** Possible structure–activity relationship of polyynes from *O. horridus* against cancer.

Polyynes^(a)^	Structure characteristics			Cancer cell lines	Activity^(b)^	References
	Carbon chain	Hydroxyl group	Double bond			
**1**	18	3	2	HCT-116, HT-29, SW-480, A549, MCF-7, MDA-MB-231, HepG2	Best	[22, 48, 49, 59]
**2**	18	3	1	HCT-116, HT-29, SW-480, A549, MCF-7, MDA-MB-231, HepG2	Better	[48, 59]
**3**	18	2	2	HCT-116, HT-29, SW-480, A549, MCF-7, MDA-MB-231, HepG2	Good	[47, 48, 59]
**4**	18	2	1	HCT-116, HT-29, SW-480, A549, MCF-7, MDA-MB-231, HepG2	Good	[47, 48, 59]
**5**	17	2	2	HCT-116, HT-29, SW-480, A549, MCF-7, MDA-MB-231, HepG2, DU145	Best	[23, 47, 48, 59]
**6**	17	2	1	HCT-116, HT-29, SW-480, A549, MCF-7, MDA-MB-231, HepG2	Better	[47, 48, 59]
**7**	17	1	2	Not available	Not available	[95–97]

^(a)^ **1**, oplopantriol A; **2**, oplopantriol B; **3**, (11*S*,16*S*,9*Z*)-9,17-octadecadiene-12,14-diyne-1,11,16-triol,1-acetate; **4**, oplopandiol acetate; **5**, falcarindiol; **6**, oplopandiol;** 7**, falcarinol. ^(b)^ Stereoselectivity also affects the activities discussed in the text.

## Supplementary Materials

Supplementary Materials Supplementary data associated with this review is the graphic abstract about bioactive polyynes from* O. horridus*.
