# Supplementary material for: Oplopanax horridus: Phytochemistry and Pharmacological Diversity and Structure-Activity Relationship on Anticancer Effects
Source: Evid Based Complement Alternat Med. 2018 Sep 13;2018:9186926. doi: 10.1155/2018/9186926 (PMC6158975; doi:10.1155/2018/9186926)
Supplement: Supplementary Materials — Supplementary data associated with this review is the graphic abstract about bioactive polyynes from O. horridus. [file 9186926.f1.pptx]

## Slide 1
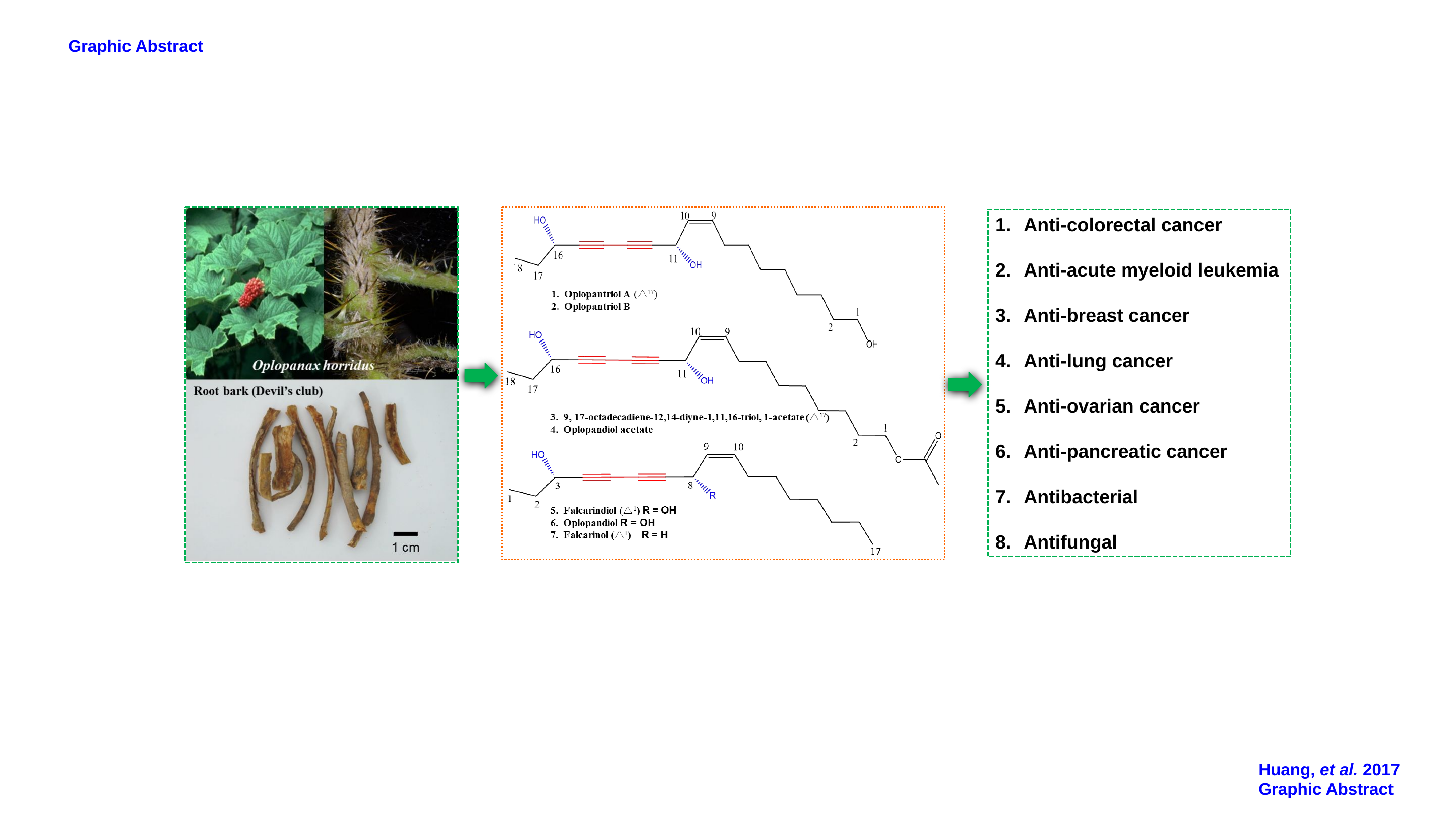

Graphic Abstract
Anti-colorectal cancer
Anti-acute myeloid leukemia
Anti-breast cancer
Anti-lung cancer
Anti-ovarian cancer
Anti-pancreatic cancer
Antibacterial
Antifungal
Huang, et al. 2017
Graphic Abstract
